# A novel linear indexing method for strings under all internal nodes in a suffix tree

**DOI:** 10.3389/fbinf.2025.1577324

**Published:** 2025-09-04

**Authors:** Anas Al-okaily, Abdelghani Tbakhi

**Affiliations:** 1 Department of Cell Therapy and Applied Genomics, King Hussein Cancer Center, Amman, Jordan; 2 Department of Pathology and Molecular Medicine, McMaster University, Hamilton, ON, Canada

**Keywords:** suffix trees, strings indexing, approximate pattern matching, reads alignment, motif search

## Abstract

Suffix trees are fundamental data structures in stringology and have wide applications across various domains. In this work, we propose two linear-time algorithms for indexing strings under each internal node in a suffix tree while preserving the ability to track similarities and redundancies across different internal nodes. This is achieved through a novel tree structure derived from the suffix tree, along with new indexing concepts. The resulting indexes offer practical solutions in several areas, including DNA sequence analysis and approximate pattern matching.

## 1 Introduction

Numerous string-processing problems arise in several scientific fields, including biology and medicine. These problems include exact and approximate pattern matching, motif search, lowest common ancestor queries, and the detection of tandem repeats. The inputs for such problems can range from small documents and databases to DNA sequences and large-scale corporate data. To address string problems more efficiently, several data structures have been designed and are commonly used, including suffix trees (Weiner, 1973; McCreight, 1976; Ukkonen, 1995), suffix arrays (Abouelhoda et al., 2004), and the FM-index (Ferragina and Manzini, 2000).

Constructing suffix trees, suffix arrays, and FM-indexes can all be achieved in linear time and space. Although building suffix trees incurs a higher constant-factor overhead than building suffix arrays and FM-indexes, their structure is more flexible and dynamic. This flexibility arises from the ability of suffix trees to identify systematic redundancies among the suffixes in the input data—capabilities not offered by suffix arrays or FM-indexes. For instance, suffix trees make it easy to observe that a subtree rooted at an internal node is isomorphic or partially isomorphic to subtrees rooted at other internal nodes. Such structural observations are not possible with suffix arrays or FM-indexes. Once these redundancies are identified and abstracted, complex string problems can be solved more efficiently than using suffix arrays, FM-indexes, or even the standard suffix tree representation.

In this work, we introduce two algorithms that index strings under all internal nodes in suffix trees in linear time and space.

## 2 Methods

Let T be a text of length n derived from an alphabet of size Σ . Let *ST* be the suffix tree of T . Let h be the height of *ST*, i.e., the maximum number of nodes between the root node and an internal node. For any internal node x in *ST*, we define the following functions: Depth(x) denotes the depth of node x , i.e., the sum of the lengths of all edges between the root of *ST* and node x ; SL(x) denotes the node to which the suffix link of node x points; SLS(x) denotes the set of nodes whose suffix links point to node x (note that for any internal node, the size of this set is up to Σ ); and Leaves(x) denotes the set of leaf nodes in the subtree rooted at node x . For any leaf node l in *ST*, Suffix_Index(l) denotes the suffix index (set during the construction of *ST*) labeled at leaf node l (if the suffix index labeled at l is i , for instance, then the label of the edges from the root node to l represents the $i^{\text{th}}$ suffix in T ).

Definition 1: Let x be an internal node, and let S(x) be the set of suffix indexes, based on T , labeled at each leaf node under x in *ST*, i.e., S(x)={Suffix_Index(l)∣l∈Leaves(x)} . Then, the suffixes *under* node x , denoted as SU(x) , are the suffix indexes in T that start from node x , i.e., SU(x)={Depth(x)+Suffix_Index(l)∣l∈Leaves(x)} .

**FIGURE 1:**
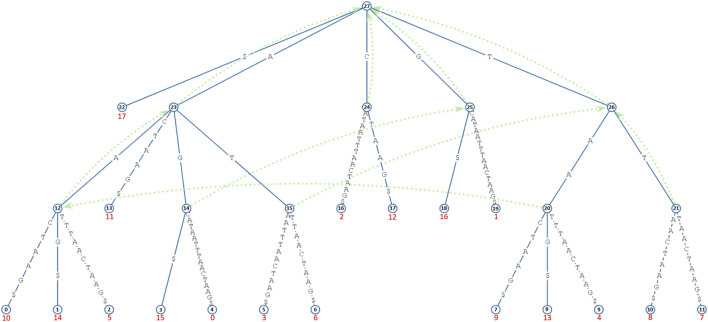
This diagram visualizes a suffix tree constructed from a string AGCCTAATTTAACTAAG$ using https://hwv.dk/st/?AGCATAATTTAACTAAG$. Each node is annotated with a unique identifier enclosed in a circle for ease of reference. The edges between nodes are labeled with substrings that represent segments of the original string along distinct suffix paths. Leaf nodes—those without children—are marked with red integers, indicating the starting positions (suffix indexes) of the corresponding suffixes in the original string. Green dotted arrows denote suffix links, which connect internal nodes according to standard suffix tree construction rules.

As an example, the S list of node 20 in Figure 1 is {9,13,4} ; therefore, SU(20)={12,16,7} since the Depth of node 20 is 3.

Observe the following properties:

- If SL(a)=b , then SU(a)⊆SU(b) . This implies that any processing or indexing assigned to suffixes in SU(a) can be implicitly applied to the same suffixes that are (and must be) in SU(b) .
- In order to achieve the above point, nodes in SLS(x) must be indexed or processed before x itself (which means that a post-order traversal is required).
- The set of suffixes that eventually need to be indexed under node x is the set of suffixes under node x minus the set of all suffixes under each node with a suffix link pointing to x , i.e., $SU\left(x\right)-\left({⋃}_{n\in SLS\left(x\right)}SU\left(n\right)\right)$ .
- The indexing process must *recursively* traverse the suffix links in *ST.*

Therefore, to compute this indexing scheme and traverse the suffix links recursively, the following tree structure must be designed and constructed.

### 2.1 Okaily-Sheehy-Huang-Rajasekaran (*OSHR*) tree structure

Given *ST*, the structure of the *OSHR* tree is defined as follows (the acronym “*OSHR*” is explained in the Acknowledgments section):

- The root node is the root of *ST.*
- There is a directed edge from node a to node b if SL(b)=a . For example, under the *OSHR* tree structure, node 25 must have a directed edge to node 14 since there is a suffix link from node 14 to node 25.
- A leaf node in the *OSHR* tree structure is any internal node v in the *ST* structure for which SLS(v)=∅ ; that is, v has no incoming suffix link (for example, node 14 in Figure 1).
- An internal node in the *OSHR* tree structure is any internal node v in the *ST* structure for which SLS(v)≠∅ ; that is, v has at least one incoming suffix link (for example, node 25 in Figure 1).
- The children of an internal node v are the nodes in SLS(v) . For example, the children of node 26 (SLS(26)) in Figure 1 are {node 15, node 21}.
- Edges have no labels.
- Leaf nodes under the *ST* structure are not included in the *OSHR* tree structure.

The directed edges in the *OSHR* tree, which are the reverse of suffix links, correspond to a simplified form of Weiner links in *ST* (as defined by Wellnitz (2021), Apostolico and Cunial (2014), Belazzougui et al. (2020). Due to the construction properties of *ST* and its suffix links, the *OSHR* tree forms a directed acyclic graph. The construction of the *OSHR* tree is carried out by traversing *ST*, and at each visited internal node v , a list called *SLS* is created at node SL(v) if it does not already exist, and v is then appended to this list. Clearly, the space and time complexities of building *OSHR* trees are both linear, O(Σn) , and this structure can be constructed either implicitly (within the *ST*) or explicitly (as a separate tree structure).

The *OSHR* tree differs from the suffix-tour graph (Starikovskaya and Vildhøj., 2015) and the suffix link tree (Starikovskaya and Vildhøj., 2015); (Apostolico and Cunial., 2014); (Belazzougui et al., 2020). Unlike the suffix-tour graph, the *OSHR* structure is acyclic. Compared to the suffix link tree, the edges in the *OSHR* tree are unlabeled, they do not include the leaf nodes of *ST*, and its leaf nodes correspond to internal nodes in *ST* that have no incoming suffix links.

### 2.2 Okaily-Tbakhi (*OT*) indexing

To identify all similarities and redundancies of strings under different internal nodes in an *ST*, a post-order traversal of the *OSHR* tree is required, during which both the *ST* and the *OSHR* tree structures are utilized.

Definition 2: We denote those strings, such as suffixes defined in the SU() function, that are present under node x (in the structure of *ST*) but not under any of the nodes in SLS(x) as the *Base Strings for node* x *or BS(x)*. Here, SLS(x) refers to the child nodes of x in the *OSHR* tree structure. The term “base” indicates that this is the first occurrence of the string under an internal node during a post-order traversal of the *OSHR* tree.

The types of strings considered under each internal node x can vary. These may include the following:

- The set of suffixes under x ( SU(x) as defined earlier). In this context, base strings are referred to as base suffixes.
- Substrings that label paths from x to each of its descendant internal nodes, defined as PU(x) . In this context, base strings are referred to as base paths.
- Specific subsets of strings.
- Strings of particular lengths

Definition 3: *OT indexing* (or *OT processing*) refers to the process of indexing or processing strings under each internal node (based on the *ST* structure, denoted as node x ) via a post-order traversal of the *OSHR* tree while avoiding the re-indexing of the same strings that have already been indexed or processed under any of the SLS(x) nodes (i.e., indexing only BS(x) ).

As a simple example, consider the task of performing *OT* indexing on the suffixes under each internal node (the set of suffixes as defined by function SU() ) in the *ST* shown in Figure 1. Let us describe the *OT* indexing process for a subset of nodes, namely, nodes 15, 21, and 26 (noting that node 26 is the parent node of nodes 15 and 21 under the *OSHR* tree structure). Before beginning the post-order traversal on the *OSHR* tree, initialize a global list called OT_index , which will store the *OT* index values for the suffixes under all internal nodes in *ST*. Now, we proceed as follows:

• Node 15:
     Since SU(15)={5,8} (suffix 5 corresponds to AATTTAACTAAG$, and suffix 8 to TTAACTAAG$) and the base suffixes at this node are also {5,8} , append 5 and 8 to OT_index (so OT_index is now equal to {5,8} ). Next, create two attributes associated with node 15: Left_OT_index=0 and Right_OT_index=1 , which correspond to the offset positions of the suffixes in the OT_index list (the suffixes for any internal node must be next to each other in the OT_index list due to the post-order traversal and the fact that $\left({⋃}_{n\in SLS\left(x\right)}SU\left(n\right)\right)\subseteq SU\left(x\right)$ ).

• Node 21:
     At this node, SU(21)={9,10} (suffix 9 corresponds to TAACTAAG$ and suffix 10 to AACTAAG$), and the base suffixes are again {9,10} , so append them to OT_index (now OT_index={5,8,9,10} ). Then, create and set Left_OT_index=2 and Right_OT_index=3 for node 21.

• Node 26:
     For this node, SU(26)={5,8,9,10,14} and the base suffixes are {14} (suffix 14 corresponds to AAG$). So, the suffix that now requires indexing is suffix 14 as the others were already indexed during the *OT* indexing process of nodes 15 and 21. Therefore, append 14 to OT_index ( OT_index is now equal to {5,8,9,10,14} ). Next, create and set Left_OT_index=0 and Right_OT_index=4 at node 26. This example illustrates how all suffixes under node 26 can be indexed through *OT* indexing without explicitly indexing each one of them.

The second part of this work introduces the concepts of base suffixes and base paths and proposes both linear and nonlinear algorithms to identify them under each internal node in the *ST*.

### 2.3 Base suffixes

We begin by defining base suffixes and then describe linear and nonlinear algorithms for finding base suffixes under each internal node in the *ST*.

Algorithm 1 Non-Trivial algorithm for identifying base suffixes.

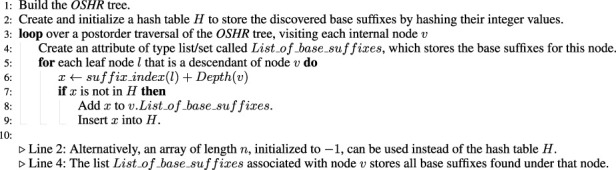

Definition 4: A *base suffix* is a suffix that occurs under an internal node in the *ST* structure, denoted as node x , and does not occur under any of the nodes in SLS(x) (the child nodes of node x in the *OSHR* tree structure). Thus, the set of base suffixes under node x (*base suffixes for node* x *or BS(x)*) is the set $SU\left(x\right)-\left({⋃}_{n\in SLS\left(x\right)}SU\left(n\right)\right)$ . If x is an *OSHR* leaf node, i.e., SLS(x)=∅ , then all suffixes under x are base suffixes.

The examples from Figure 1 help illustrate the concept of base suffix. The base suffixes for node 26 are the set 14 (base suffix 14 corresponds to AAG$). The base suffixes for node 23 are {11, 15, 6, 1, 4} (base suffix 11 corresponds to ACTAAG$, 15 to AG$, 6 to ATTTAACTAAG$, 1 to GCATAATTTAACTAAG$, and 4 to TAATTTAACTAAG$). Because node 20 has no incoming suffix links (SLS(20)=∅) , all suffixes under it are base suffixes, namely, {12, 16, 7} (base suffix 12 corresponds to CTAAG$, 16 to G$, and 7 to TTTAACTAAG$). Node 12 has no base suffixes as all suffixes under it are already covered under nodes of SLS(12) ( SLS(12)={node 20} ).

Definition 5: If bs is a base suffix under node x , then the *extended suffixes* of bs are all suffixes identical to bs that occur under each ancestor of x (where ancestry is defined according to the *OSHR* tree structure).

For example, suffix 8 is a base suffix for node 15 (corresponds to TTAACTAAG$, starting from node 15 and ending at leaf node 6). The extended suffixes corresponding to this base suffix are the occurrences of TTAACTAAG$ under node 26 (ending at leaf node 11) and under the root node (ending at leaf node 10).

Observation 1: Based on definitions 4 and 5, the upper bound on the number of extended suffixes for any base suffix is O(h) , where the last extended suffix of any base suffix is the one occurring under the root node.

Observation 2: Based on definitions 4 and 5 and Observation 1, the base suffixes under all internal nodes in *ST* must be n distinct integers ranging from 0 to n−1 (i.e., indexes of all suffixes in T ).

In the example provided in Section 2.2, once the traversal reaches the root node, the OT_index list will encompass all n base suffixes, ordered as identified through the post-order traversal of the *OSHR* tree. Consequently, the root node must have a Left_OT_index of 0 and a Right_OT_index of n−1 .

Therefore, once a base suffix is processed or indexed, this processing or indexing can be applied *implicitly* to all O(h) extended suffixes throughout the post-order traversal of the *OSHR* tree. So, what will be processed or indexed *explicitly* is each of the n base suffixes. As a result, *OT* indexing or processing of all suffixes under all internal nodes in the *ST* can be achieved with a complexity factor of n .

Algorithm 2 Non-Trivial algorithm for identifying base suffixes.

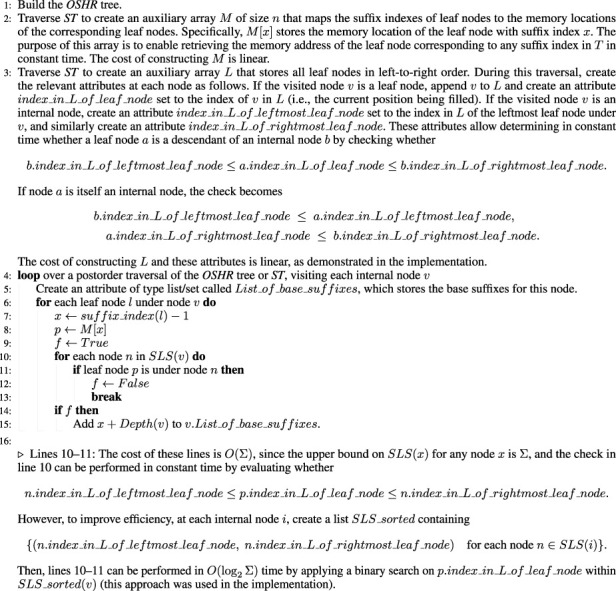

Algorithm 3 Linear algorithm for finding base suffixes.

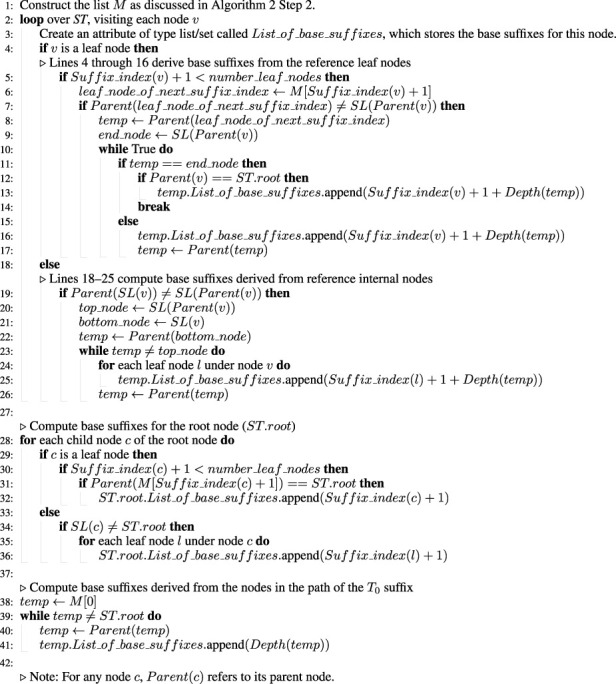

#### 2.3.1 Finding base suffixes

To find base suffixes under each internal node in *ST*, we present four approaches: a trivial algorithm with O(nh) complexity, a non-trivial algorithm with O(nh) complexity but more time-efficient than the trivial algorithm, a second non-trivial algorithm with $O\left(nh\log_{2}\Sigma \right)$ complexity, and, finally, a linear algorithm.

Trivially, all base suffixes under each internal node can be identified using the following algorithm. Build the *OSHR* tree (to mainly generate the SLS lists for each internal node). Next, traverse the *OSHR* or *ST* tree where at each visited node v : create a hash table for the set ${⋃}_{n\in SLS\left(v\right)}SU\left(n\right)$ ; then, check whether each suffix in SU(v) exists in the hash table; if not, then that suffix is considered a base suffix for (under) node v . The cost of this algorithm is O(nh) .

Given Observation 2, the following non-trivial algorithm, which requires auxiliary O(n) space (for a hash table named H ), will cost O(nh) but is clearly more time-efficient than the trivial algorithm. The algorithm is stated in Algorithm 1. Briefly, during the post-order traversal of the *OSHR* tree, check at each visited internal node v whether each suffix in SU(v) is already in H ; if not, then it is a base suffix for (under) node v and add this base suffix (as a number) into H .

The second non-trivial algorithm achieves $O\left(nh\log_{2}\Sigma \right)$ time complexity using O(n) auxiliary space, as shown in Algorithm 2. After building the *OSHR* tree, traverse the *OSHR* or *ST* tree, and at each visited node v : loop through each leaf node (let the leaf node be l ) under node v , then check whether the leaf node labeled with suffix index equal to Suffix_Index(l)−1 is a descendant node under any node in SLS(v) ; if not, then that suffix is considered a base suffix for (under) node v . In the naive approach, the cost of checking whether node l is a descendant node under any node in SLS(v) is O(Σ) as the upper bound for the SLS() list for any internal node is Σ , but with a simple trick (which was also implemented), the cost can be reduced to $O\left(\log_{2}\Sigma \right)$ .

The linear algorithm was motivated by Observation 2. As the total number of base suffixes across all internal nodes in *ST* is equal to n , if each base suffix can be found in constant time, the total cost will be O(n) . To achieve this, two definitions must be introduced.

Definition 6: Let A be a leaf node in *ST* with suffix index x and B be the parent of A . Let C be the leaf node with suffix index x+1 and D be the parent of C . If SL(B)≠D , we call each node between C and D an *inbetween node* for A , and we call A a *reference leaf node* for each of those inbetween nodes.

As shown in Figure 1, node 6 is a reference leaf node for node 21 and node 21 is an inbetween node for node 6.

Note that a reference leaf node can be associated with O(h) inbetween node, and an inbetween node can correspond to O(Σ) reference leaf nodes. Additionally, the total number of reference leaf nodes across all internal nodes in *ST* is much fewer than n .

Definition 7: Let A be an internal node in *ST*, with parent B . Let SL(A)=C , and let D be the parent of C . If SL(B)≠D , we call each node between C and D an *inbetween node* for A , and we call A a *reference internal node* for each of those inbetween nodes.

As illustrated in Figure 1, node 20 is a reference internal node for node 23 and node 23 is an inbetween node for node 20.

A reference internal node may have O(h) inbetween nodes, and an inbetween node can correspond to O(Σ) reference internal nodes. Moreover, an inbetween node may be associated with O(Σ) reference leaf nodes and O(Σ) reference internal nodes. Finally, the total number of reference internal nodes across all internal nodes in *ST* is much fewer than n .

The linear algorithm derives and identifies each base suffix in constant time using the inbetween nodes, reference leaf nodes, and reference internal nodes as stated in Algorithm 3. Since the upper bound on the number of reference leaf nodes and reference internal nodes is O(Σ) for any internal node (most internal nodes are *not* inbetween nodes), the cost for finding these nodes is O(Σn) . In addition, computing each of the n base suffixes has a cost of O(1) , as shown in Algorithm 3. Therefore, the total cost is O(Σn) .

Theorem 1. Finding all base suffixes under all internal nodes in a *ST* can be achieved in linear time and space $\left(O\left(\Sigma n\right)\right)$ .

Once the base suffixes have been identified for each internal node in an *ST* in linear time, let us *OT* index the n base suffixes using an indexing operation P , where the cost of P is p ; then, the total cost for *OT* indexing all n base suffixes will be O(pn) . Since the *OT* indexing process of each base suffix will be implicitly applied to each of its O(h) extended suffixes, then the total cost of applying process P to all suffixes under all internal nodes in an *ST* is also O(pn) (as opposed to O(pnh) ).

After finding the base suffixes under all internal nodes in an *ST* in linear time, several applications become feasible, particularly when combined with *OT* indexing. One such application is illustrated by the following example.

Let the *OT* indexing of the base suffixes in an *ST* be applied to solve the problem of exact pattern matching (which is a fundamental problem in biological applications such as read alignment, motif search, and genome annotation). Suppose there is a pattern that exactly matches one of the base suffixes under some node v . In this case, the final *OT* index (constructed across the entire *ST*) can be used to determine that the pattern has an exact match under node v (the matching here is with the base suffix itself) and also under every ancestor node of v (with ancestry based on the *OSHR* tree structure), where the pattern’s exact match corresponds to the extended suffix (of the base suffix) under each ancestor node. This is achieved by explicitly applying *OT* indexing only on the base suffix under node v , while the extended suffixes under the ancestor nodes of v are implicitly *OT*-indexed through the post-order traversal of the *OSHR* tree (as described in the *OT* indexing example).

### 2.4 Base paths

The motivation for this indexing approach arises from the following observations. First, the primary source of complexity in a tree structure lies in the branching caused by internal nodes. Second, the tails of suffixes (i.e., the labels between a leaf node and its parent) are often very long, making their processing computationally expensive. Third, if a process reaches an internal node whose children are all leaf nodes, the computational cost for handling these leaves is bounded by the alphabet size Σ . Consequently, instead of explicitly indexing or processing the full suffix tails, it is generally sufficient (and more efficient) to process only the labels along the paths connecting internal nodes to their descendant *internal* nodes.

Next, we define the concept of base paths and present algorithms for identifying base paths under each internal node in an *ST*, with both linear and nonlinear costs.

Algorithm 4 Non-trivial algorithm for finding base paths.

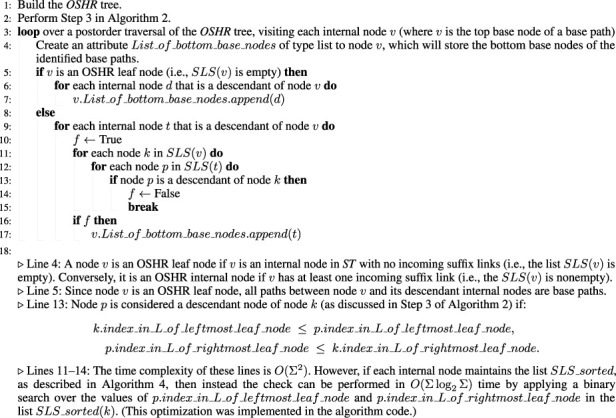

Definition 8: Let x be an internal node, and let PU(x) be the set of internal descendant nodes under x in an *ST*. A *base path* is a path between two internal nodes, for example, nodes A and B , such that this path does not occur between two other internal nodes C and D , where SL(C)=A , SL(D)=B , and D is a descendant node of C . Thus, the set of base paths under node x (base paths for node x or BP(x) ) is the set $\left\{PU\left(x\right)-\left\{SL\left(x\right)|x\in {⋃}_{n\in SLS\left(x\right)}PU\left(n\right)\right\}\right\}$ . Note that if an internal node x is an *OSHR* leaf node (SLS(x)=∅) , then all the paths between the node and its descendant internal nodes are base paths. If the path between node A and node B is a base path, then node A is called the *top base node* and node B is the *bottom base node*.

For example, in Figure 1 (noting that the T string for the suffix tree is relatively short), the set of base paths under node 23 is {node 12, node 14}. Similarly, for node 26, it is {node 20, node 21}. For the root node, the set is {node 14, node 15, node 24, node 20, node 21}.

Definition 9: If bp is a base path between a top base node A and a bottom base node B , then the path between node SL(A) and node SL(B) is called an *extended path* of bp . This relationship extends recursively to all paths between the ancestor nodes of A and the ancestor nodes of B in the *OSHR* tree structure via suffix links.

For instance, the path between the root and node 25 is an extended path of the base path between nodes 23 and 14.

Observation 3: Based on definitions 8 and 9, the upper bound on the number of extended paths for any base path is O(h) , where the last extended path for bp is the one whose top base node is the root node.

Observation 4: Based on definitions 8 and 9 and Observation 3, any path from the root node to an internal node can be the final extended path of a base path; hence, the total number of base paths is bounded by Σn .

Algorithm 5 Linear algorithm for finding base paths.

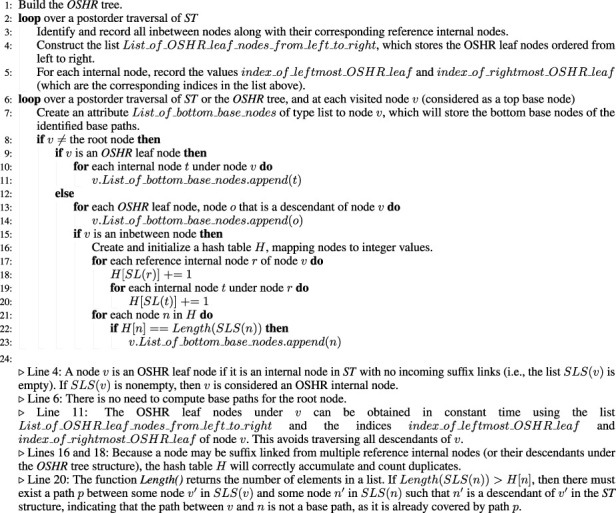

### 2.6 Finding base paths

All base paths in an *ST* can be identified using a straightforward (trivial) algorithm with time complexity O(nh) , described as follows. The algorithm starts by building the *OSHR* tree, followed by a post-order traversal of the *OSHR* tree or *ST*. At each visited node v , it constructs a hash table containing the set $SL\left(x\right)|x\in {⋃}_{n\in SLS\left(x\right)}PU\left(n\right)$ . Then, for each descendant internal node d under v , if d is not in the hash table, the path between v and d is identified as a base path. In contrast, this work introduces a non-trivial algorithm that improves upon the trivial algorithm, with a time complexity of $O\left(nh\Sigma \log_{2}\Sigma \right)$ , and a linear algorithm with both time and space complexity of O(Σn) .

Algorithm 4, which is analogous to Algorithm 2, can find base paths under all internal nodes with a time complexity of $O\left(nh\Sigma \log_{2}\Sigma \right)$ and a space complexity of O(Σn) . The algorithm starts by building the *OSHR* tree and traverses the *OSHR* tree or *ST*, and at each visited node v : traverse each descendant internal node under v (say, node d ) and check whether any node in SLS(d) is a descendant of any node in SLS(v) ; if not, the path between v and d is a base path. This check has a worst-case cost of $O\left(\Sigma^{2}\right)$ as the maximum size of any SLS set is Σ , but with a simple trick (which was also implemented), the cost can be reduced to $O\left(\Sigma \log_{2}\Sigma \right)$ .

Since the total number of base paths is no more than Σn as given in Observation 4 and if the time cost for finding each base path is constant, all base paths can be found in linear time and space, O(Σn) . This is precisely what Algorithm 5 achieves by leveraging the properties of the *OSHR* tree and reference internal nodes (Definition 7).

Theorem 2: All base paths under all internal nodes in an *ST* can be found in linear time and space O(Σn) .

Once base paths are computed for each internal node in an *ST*, any index or process P with cost p applied to a base path t under an internal node will implicitly apply to the O(h) extended paths of t . Therefore, the total cost of applying process P for all paths under all internal nodes in an *ST* will be proportional to n , costing O(np) instead of O(phn) .

The following is an example of *OT* indexing base paths. Let the *OT* index be constructed to resolve the pattern matching problem, as discussed in the example at the end of Section 2.3.1, where the pattern here is an exact match of one of the base paths under node v . Then, the *OT* index (constructed across the entire *ST*) can be used to determine that the pattern has an exact match under node v (here, the matching is with the base path itself) and also under every ancestor node of v (the ancestry is based on the *OSHR* tree structure), where the exact match is the extended path (of the base path) under each ancestor node.

## 3 Results

To assess the correctness and effectiveness of the proposed algorithms, we evaluated them on the genomes of the following organisms, with genome sizes ranging from ∼ 1 Mb to ∼ 100 MB): WS1 bacterium JGI 0000059-K21 (bacteria, 0.5 MB), *Astrammina rara* (protist, 1.5 MB), *Nosema ceranae* (fungus, 5.5 MB), *Cryptosporidium parvum Iowa II* (protist, 8.8 MB), *Spironucleus salmonicida* (protist, 12.5 MB), *Tieghemostelium lacteum* (protist, 22.8 MB), *Fusarium graminearum PH-1* (fungus, 35.5 MB), *Salpingoeca rosetta* (protist, 54 MB), and *Chondrus crispus* (Algae, 102.5 MB).

In the preprocessing step, header lines and newline characters were removed from each FASTA file, and all lowercase nucleotides were converted to uppercase. As a result, each genome was converted to a single-line sequence with all nucleotides in uppercase. The Python script used for this preprocessing step is available at the repository: https://github.com/aalokaily/Finding_base_suffixes_and_base_paths_in_suffix_trees.

All five algorithms presented in this study were implemented in Python and are publicly available in the aforementioned repository. Notably, the non-trivial algorithm (Algorithm 2) was excluded from the comparative analysis because it is both theoretically and empirically slower than the other non-trivial algorithm (Algorithm 1), as demonstrated by preliminary tests (data not shown). Regarding base suffix identification, the results obtained using the linear algorithm (Algorithm 3) perfectly matched those of its non-trivial counterpart (Algorithm 1) for each internal node in the *ST*. Across all tested genomes, the total number of base suffixes under all internal nodes is equal to n . Similarly, for base path identification, the outputs of the non-trivial algorithm (Algorithm 4) and the linear algorithm (Algorithm 5) were identical across all internal nodes in an *ST*. Across all tested genomes, the total number of base paths remained bounded by O(Σn) . A summary of these results is provided in Table 1.

**TABLE 1:** Results from the evaluation and comparison of algorithms 1 and 3 (for base suffix identification) and algorithms 4 and 5 (for base path identification).

Genome	WS1 bacterium	Astrammina	Nosema	Cryptosporidium	Spironucleus	Tieghemostelium	Fusarium	Salpingoeca	Chondrus
GenBank accession no.	GCA000398605.1	GCA000211355.2	GCA000988165.1	GCA000165345.1	GCA000497125.1	GCA001606155.1	GCF000240135.3	GCA000188695.1	GCA000350225.2
No. of alphabets	5	5	4	15	5	4	5	5	5
No. of nucleotide/leaf nodes	509,552	1,450,096	5,690,749	9,102,325	12,954,589	23,375,663	36,458,047	55,440,310	104,980,421
No. of internal nodes	328,917	926,087	3,849,880	5,931,181	8,684,514	15,606,221	22,972,065	37,480,799	81,909,252
No. of OSHR leaf nodes	132,774	380,518	1,353,289	2,328,480	3,127,031	5,740,807	9,800,385	11,545,032	15,307,982
No. of OSHR internal nodes	196,143	545,569	2,496,591	3,602,701	5,557,483	9,865,414	13,171,680	25,935,767	66,601,270
Algorithm 1 (time complexity/sec)	2,409,867/3	6,838,684/9	26,549,891/37	42,989,315/61	60,212,775/92	109,641,901/172	172,751,172/278	250,969,523/446	457,348,511/983
Algorithm 3 (time complexity/sec)	6,117,025/3	18,036,912/8	83,860,995/41	186,083,328/113	215,252,439/110	364,737,747/209	851,871,768/561	19,667,387,766/8,763	2,787,481,705/1,508
No. of base paths	1,229,296	3,685,339	16,277,962	27,440,361	37,673,155	73,606,066	116,915,929	161,201,632	234,284,261
Algorithm 4 (time complexity/sec)	6,314,996/18	18,734,271/59	99,497,879/300	210,245,283/698	238,310,327/731	430,212,888/1,310	804,854,850/3,463	15,946,058,214/79,911	3,670,449,444/12,091
Algorithm 5 (time complexity/sec)	3,930,963/6	11,316,346/24	47,510,898/85	76,040,038/135	108,340,936/210	200,650,145/389	307,254,707/630	474,007,996/1,027	879,529,459/3,522

**FIGURE 2:**
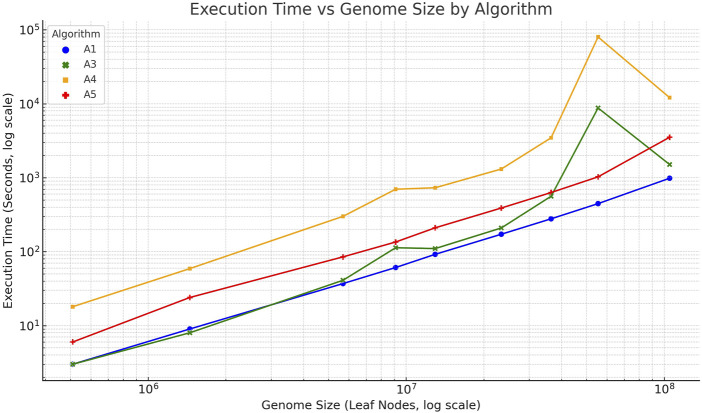
Execution time (log–log scale) of algorithms 1 (blue), 3 (green), 4 (orange), and 5 (red) plotted against genome size (number of nucleotide/leaf nodes). The linear trend observed for algorithms 3 and 5 confirms their linear-time behavior, while algorithms 1 and 4 exhibit superlinear growth.

Finally, a statistical analysis was conducted to evaluate the scalability and performance differences among the proposed algorithms. The execution time for each algorithm was plotted against the genome size, as shown in Figure 2. Linear regression confirmed a strong linear relationship between genome size and runtime for linear algorithms 3 and 5 $\left(R^{2}>0.99\right)$ , consistent with theoretical expectations. In contrast, algorithms 1 and 4 exhibited superlinear growth due to their dependence on variable h values. One-way ANOVA showed significant differences in runtime across all algorithms (F=…,p<0.001) . *Post hoc* pairwise t-tests (Bonferroni-corrected) confirmed that Algorithms 3, 5 were significantly faster than their non-trivial counterparts (algorithms 1 and 4, respectively; p<0.01 ). These findings empirically validate the linear time performance of the proposed linear algorithms (Algorithms 3, 5) across genomes of varying sizes.

## 4 Conclusion

The primary contribution of the *OT* indexing of base suffixes and base paths is their linear time and space cost for indexing all suffixes and paths under all internal nodes in an *ST*. This property is not achievable using existing suffix tree construction algorithms (such as Ukkonen’s algorithm (Ukkonen., 1995) or McCreight’s algorithm (McCreight, 1976)) or other approaches related to suffix trees. The resulting linear *OT* index enables indexing all suffixes or paths under all internal nodes with a complexity factor of n instead of nh . This capability can be incorporated into more efficient solutions for problems related to next-generation sequencing analysis (Li, 2013; Hu et al., 2024; Guo et al., 2024; Wang et al., 2024a; Wang et al., 2024b) and machine learning (Zhao et al., 2025; Yue et al., 2024a; Zhao et al., 2024; Zhao et al., 2022; Yue et al., 2024b).

## Data Availability

Source code of the algorithms are available at https://github.com/aalokaily/Finding_base_suffixes_and_base_paths_in_suffix_trees. Further inquiries can be directed to the corresponding author.
